# A New Perspective for the Training Assessment: Machine Learning-Based Neurometric for Augmented User's Evaluation

**DOI:** 10.3389/fnins.2017.00325

**Published:** 2017-06-13

**Authors:** Gianluca Borghini, Pietro Aricò, Gianluca Di Flumeri, Nicolina Sciaraffa, Alfredo Colosimo, Maria-Trinidad Herrero, Anastasios Bezerianos, Nitish V. Thakor, Fabio Babiloni

**Affiliations:** ^1^Department of Molecular Medicine, Sapienza Università di RomaRome, Italy; ^2^BrainSigns srlRome, Italy; ^3^Neuroelectrical Imaging and BCI Lab, Fondazione Santa Lucia (IRCCS)Rome, Italy; ^4^Department of Anatomical, Histological, Forensic, and Orthopedic Sciences, Sapienza Università di RomaRome, Italy; ^5^Clinical and Experimental Neuroscience (NiCE-IMIB), School of Medicine, Institute of Aging Research, University of MurciaMurcia, Spain; ^6^Centre for Life Sciences, Singapore Institute for Neurotechnology, National University of SingaporeSingapore, Singapore

**Keywords:** training assessment, human factor, brain activity, EEG, machine learning, human machine interaction

## Abstract

Inappropriate training assessment might have either high social costs and economic impacts, especially in high risks categories, such as Pilots, Air Traffic Controllers, or Surgeons. One of the current limitations of the standard training assessment procedures is the lack of information about the amount of cognitive resources requested by the user for the correct execution of the proposed task. In fact, even if the task is accomplished achieving the maximum performance, by the standard training assessment methods, it would not be possible to gather and evaluate information about cognitive resources available for dealing with unexpected events or emergency conditions. Therefore, a metric based on the brain activity (*neurometric*) able to provide the Instructor such a kind of information should be very important. As a first step in this direction, the Electroencephalogram (EEG) and the performance of 10 participants were collected along a training period of 3 weeks, while learning the execution of a new task. Specific indexes have been estimated from the behavioral and EEG signal to objectively assess the users' training progress. Furthermore, we proposed a neurometric based on a machine learning algorithm to quantify the user's training level within each session by considering the level of task execution, and both the behavioral and cognitive stabilities between consecutive sessions. The results demonstrated that the proposed methodology and neurometric could quantify and track the users' progresses, and provide the Instructor information for a more objective evaluation and better tailoring of training programs.

## Introduction

More than 80 years ago, Spearman (1928) stated that psychological writings “crammed” a lot of allusions to human error in an “incidental manner,” but “they hardly arrived at considering such concept systematically and profoundly.” The past three decades have seen an increasing interest in studying the *Human Factor* (HF) in working environments, especially its causes and possible prevention approaches. Until the 90s, a major objective of the scientific community was to limit the human contribution to the conspicuously catastrophic breakdown of high hazard enterprises such as air, sea, and road transports, nuclear power generation, and chemical process plants. Accidents in those operative environments might cost many lives, create widespread environmental damage, and generate public and political concerns. In this regard, due to its high safety standards, the aviation domain was of great interest. In fact, aircraft accident investigations had revealed that 80% of accidents were based on human error, but further investigation indicated that a significant portion of human error was attributable to HF failures primarily associated with inadequate communication and coordination within the crew (Taggart, 1994). Beyond the technology and equipment progress, specific HF training methods (e.g., *Crew Resources Management*—CRM) have led to the reductions of aviation accidents. In fact, since the end of 90s, CRM training has been required for all military and commercial US aviation crews and air couriers (Helmreich, 1997). Some aviation HF programs have been adapted to the healthcare field with the aim to improve teamwork in healthcare and to reduce the error commission probability. Numerous reports, scientific meetings, and publications have continued to seek solutions to improve safety, and many of them have identified the personnel training as a significant strategy in achieving such a goal (Barach and Small, 2000; Leonard and Tarrant, 2001; Barach and Weingart, 2004; Hamman, 2004; Leonard et al., 2004). *Training* refers to a systematic approach to learning and development to improve individual, team, and organizational effectiveness (Goldstein and Ford, 2002). The importance and the interest in the concept of training in operative environments (e.g., aviation, hospital, public transport) is reflected by the regular publication of scientific reviews in the *Annual Review of Psychology* since 1971 (Campbell, 1971; Goldstein, 1980; Wexley, 1984; Tannenbaum and Yukl, 1992; Salas et al., 2001; Aguinis and Kraiger, 2009). Training not only could result in the acquisition of new skills (Hill and Lent, 2006; Satterfield and Hughes, 2007) but also in improved declarative knowledge, enhance strategic knowledge, defined as knowing when to apply a specific knowledge or skill, in particular during unexpected events (Kozlowski et al., 2001; Borghini et al., 2015). Furthermore, despite the time passed from the last training session, there is also the need to assess if the operator is still able to work ensuring a high performance level, hence, a proper level of safety. For such a reason, another issue is the necessity of objectively monitoring and assessing operators' performance (Leape and Fromson, 2006), especially in terms of cognitive resource and brain activations (Di Flumeri et al., 2015). For example, during the training courses, it could be possible to obtain a series of measures of the operator's performance by using simulators (Cronin et al., 2006). These could be used as part of an ongoing certification process to ensure that operators will be able to maintain their knowledge and skills, identify areas of weakness, and promptly react in order to avoid possible risks (Astolfi et al., 2012; Broach, 2013). Nevertheless, although the results in terms of performance should be the same, the cognitive demand for the same operator could be not. In other words, after a certain time the operator should still be able to execute the same task by achieving the same performance level, but it might require different amount of cognitive resources. Therefore, different operators could achieve the same results, but involving a different amount of cognitive resources.

Nowadays one of the current limitations of the standard training assessment procedures is indeed the lack of objective information about the amount of cognitive resources requested by the trainees during the operative activity. The difference between the available cognitive resources and the amount of those involved for the task execution is called *Cognitive Spare Capacity* (Borghini et al., 2014, 2015; Vecchiato et al., 2016). The higher the cognitive spare capacity during a normal working activity is (i.e., the operator is involving a low amount of cognitive resources), the greater the operator ability to perform secondary tasks or to react to unexpected—emergency events is.

Therefore, the proposed work aimed to use neurophysiological signals (i.e., EEG) to provide additional objective information regarding the progresses of a trainee throughout the training program, on the base of the current brain activations with respect to previous training sessions. Such concern is based on the experimental hypothesis that, during a training period the execution of the task become more automatic and less cognitive resources are required, thus higher amount of cognitive resources will be available. In other words, when the users are not trained, the pattern of brain activations should change any time they execute the task. On the contrary, when the users become well-trained, the task performance reach the saturation area, and the brain's patterns become stable as well. Therefore, the idea presented in this work was to use a machine learning approach to track changes in the user's brain features along 3 weeks of training. The expected result was to note a *plateau* of the classifier performance once the user's brain patterns become stable across consecutive training sessions, in other words, when the user should be defined “cognitively” trained.

Under the recognition of the user's mental states, machine learning techniques are able to extract the most significant characteristics (brain features) closely related to the examined mental status from the big amount of neurophysiological data. A diverse array of machine learning algorithms has been developed to cover the wide variety of applications and issues exhibited across different machine learning problems (Murphy et al., 2012). For example, machine learning algorithms have been used for *Brain Computer Interface* (BCI) study (Parra et al., 2003; Aricò et al., 2014; Schettini et al., 2014; Wu et al., 2014; Marathe et al., 2016), mental states evaluation such as vigilance (Shi and Lu, 2013), arousal (Wu et al., 2014), alertness (Lin et al., 2006), drowsiness (Lin et al., 2005), EEG temporal feature evaluation as the *error related negativity*—ERN (Parra et al., 2003), or emotions (Li and Lu, 2009; Lin et al., 2010; Wang et al., 2014) and cognitive control behavior assessment (Borghini et al., 2017). Machine learning algorithms vary greatly. Attempts to characterize machine learning algorithms have led to blends of statistical and computational theories in which the goal is to characterize simultaneously the sample complexity (how much data are required to calibrate accurately the algorithm) and the computational complexity (how much computational effort is required; see Decatur et al., 2000; Chandrasekaran and Jordan, 2013; Shalev-Shwartz and Zhang, 2013). In this regards, we used the *automatic stop stepwise linear discriminant analysis* (asSWLDA, Aricò et al., 2016a) algorithm, since it is able to address important issues for the application of machine learning algorithms across different days (Aricò et al., 2016b). In particular, the asSWLDA can avoid both the *under*- and *over-fitting* issue in the feature selection phase, and it does not require any calibration up to a month, since the asSWLDA showed high performance stability and reliability over time for mental workload evaluation.

Therefore, the objectives of our study were to investigate (i) the advantages of the neurophysiological measures as support for an objective training assessment with respect to the standard performance evaluation, and (ii) to provide a possible neurometric for tracking and quantifying the user's training level within each session, and across the different sessions by using a machine learning approach.

## Materials and methods

### Characterization of learning processes

Experimental evidences suggest that motor memory formation occurs in two subsequent phases (Karni et al., 1994; Armitage, 1995; Dudai, 2004; Luft and Buitrago, 2005). The first is the initial encoding of the experience during training that occurs within the first minutes-to-hours after training, and it is characterized by rapid improvement in performance. The second phase is the memory consolidation, and involves a series of systematic changes at the molecular level, that occur after training. This second phase requires longer time. The literature dealing with the effect of practice on the functional anatomy of task performance is extensive and complex, comprising a wide range of papers from disparate research perspectives (Chein and Schneider, 2005; Doyon and Benali, 2005; Parsons et al., 2005; Erickson et al., 2007; Dux et al., 2009; Wiestler and Diedrichsen, 2013; Parasuraman and McKinley, 2014; Sampaio-Baptista et al., 2014; Borghini et al., 2016). Across these studies, three main patterns of practice-related activation change can be distinguished. Practice may result in an increase or a decrease in activation in the brain areas involved in task performance, or it may produce a functional reorganization of brain activity, which is a combined pattern of activation increases and decreases across a number of brain areas (Kelly and Garavan, 2005). Activations seen earlier in practice involve generic attentional and control areas, especially the *Prefrontal Cortex* (PFC), the *Anterior Cingulate Cortex* (ACC) and the *Posterior Parietal Cortex* (PPC). It has been observed as a change in the location of activations is associated with a shift in the cognitive processes underlying task performance (Poldrack, 2000; Glabus et al., 2003). In other words, with practice the task-related processes fall away and there is a shift from controlled (mainly pre-frontal and frontal brain areas) to automatic processes (mainly parietal brain areas).

It has been demonstrated that the most important cognitive processes involved in learning are working memory, attention, procedural memory, information processing, adaptive control, and long-term memory access (Shiffrin and Schneider, 1977; Logan, 1988; Shadmehr and Holcomb, 1997; Petersen et al., 1998; Bernstein et al., 2002; Ridderinkhof et al., 2004; Kelly and Garavan, 2005; Gluck and Pew, 2006; Estes, 2014). As quoted previously, the frontal and parietal brain regions appear to create a robust network and to be the most cooperative ones during learning progress. In fact, frontal regions are essential for organizing on-line corrections in response to unexpected events, or they become activated in novel situations (Mutha et al., 2011). Parietal regions, instead, may cover the process of learning and/ or storing new visuo-motor associations leading to error reduction through adaptation (Diedrichsen et al., 2005). Moreover, once learning has occurred, parietal regions may also store the set of knowledge required to overcome eventual mismatch with respect to the plan (the so called “target jump”; Desmurget et al., 1999; Pisella et al., 2000; Gréa et al., 2002). For such reasons, the brain features selected to define a neurophysiological metric usable to give information from a cognitive point of view during a training program were the frontal and parietal theta, and the frontal and parietal alpha EEG rhythms.

In fact, one of the most prominent neurophysiological events linked to the increase of information processing, working memory, decision making process, and sustained attention (Botvinick et al., 2004; Mitchell et al., 2008) is the increase (e.g., *synchronization*) of the theta activity over the prefrontal and frontal brain areas (Berka et al., 2007; Berka and Johnson, 2011; Galán and Beal, 2012; Jaušovec and Jaušovec, 2012; Borghini et al., 2013, 2016; Mackie et al., 2013; Cartocci et al., 2015). Additionally, frontal theta synchronization has also been demonstrated to be correlated with memory load (Jensen and Tesche, 2002), task difficulty (Gevins et al., 1997; Aricò et al., 2015), error processing (Luu et al., 2004), and recognition of previously viewed stimuli (Arrighi et al., 2016).

Similarly, studies of spatial memory have reported increased parietal theta activity during learning (Kahana et al., 1999; Caplan et al., 2001, 2003; Sauseng et al., 2005; Jacobs et al., 2006; Gruzelier, 2009). In fact, several lines of evidence suggest that parietal theta oscillations play an important role in memory formation, and are thought to play a critical role in the induction of long-term plasticity, associated with memory consolidation (Caplan et al., 2003; Buzsáki, 2005; Anderson et al., 2010; Benchenane et al., 2010; Kropotov, 2010; Nieuwenhuis and Takashima, 2011; Chauvette, 2013).

Concerning the alpha EEG band, numerous studies have suggested that alpha is associated with the cognitive functions of attention (Klimesch, 2012), perception (Di Flumeri et al., 2016), long-term memory (LTM; Jensen et al., 2002; Klimesch, 2012; Toppi et al., 2014), and working memory (WM, Garavan et al., 2000; Jensen et al., 2002; Sauseng et al., 2005; Gruzelier, 2009; Borghini et al., 2016). Fairclough et al. (2005) found that the sustained response to a demanding task produced alpha suppression, and more recently Jaušovec and Jaušovec (2012) investigated the influence of WM training on intelligence and brain activity: they found out that the influence of WM training on patterns of neuroelectric brain activity was most pronounced in the theta and alpha bands (theta band synchronization was accompanied by alpha desynchronization), and hence concluded that WM training increased individuals performance on tests of intelligence. Furthermore, Klimesch (2012) proposed that alpha activity has both roles of task-irrelevant networks inhibition and timing within task relevant networks. Alpha activity thus plays an important role for attention by supporting processes within the attentional focus and blocking processes outside its focus. The two fundamental functions of attention as filter (suppression and selection) enable selective access to the *Knowledge System* (KS) and operate according to the proposed inhibition timing function of alpha-band activity (Klimesch et al., 2011). Thus, periods of prolonged access should be associated with ERS, reflecting increased (i.e., synchronization) alpha-band activity (Benedek et al., 2011; Zanto et al., 2011; Jauk et al., 2012).

### Experimental group

The experiment was conducted following the principles outlined in the Declaration of Helsinki of 1975, as revised in 2000. It received the favorable opinion from the Ethical Committee of the *National University of Singapore* (NUS), Centre for Life Sciences (NUS-IRB Ref. No: 13-132, NUS-IRB Approval No: NUS 1864). The study involved only healthy, normal subjects, recruiting on a voluntary basis. Informed consent was obtained from each subject on paper, after the explanation of the study. The selection of the participants has been done accurately in order to ensure the homogeneity of the experimental sample. Ten healthy volunteers (students of the *National University of Singapore*—NUS) have given their informed consent for taking part at the experiment and each of them has been paid SG$200 to attend the whole experimentation.

### NASA—Multi Attribute Task Battery (MATB)

**Figure 1:**
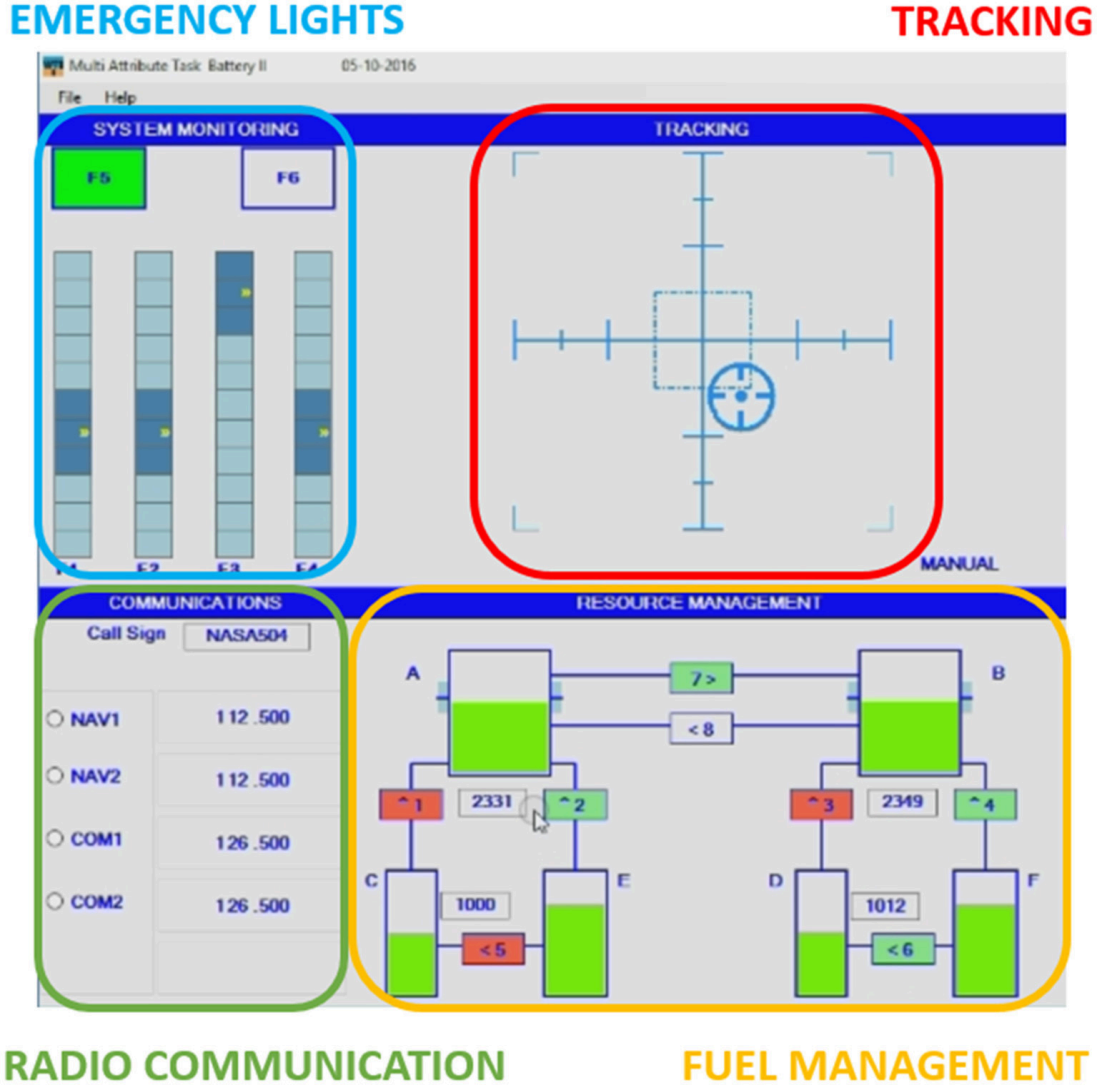
NASA *Multi Attribute Task Battery* (MATB) interface. Emergency lights task (SYSM) in the blue rectangle; Tracking task (TRCK) in the red rectangle; Radio Communication (COMM) in the green rectangle, and Fuel managing task (RMAN) in the orange rectangle (A–F are the fuel tanks).

The NASA—*Multi Attribute Task Battery* (MATB, Comstock and Arnegard, 1992) is a computer-based task designed by the NASA to evaluate the operator task performance and workload during the execution of multi-tasks (Figure 1). It could be freely download from the NASA website at the following address: http://matb.larc.nasa.gov/. The MATB is a platform for the evaluation of the cognitive operational capability, since it could provide different tasks that have to be attended by the subject in parallel, and each task could also be modulated in difficulty. By such capabilities, it is possible to simulate many of the modern operative works (e.g., piloting an airplane, medical surgery) therefore to investigate different cognitive phenomena in operative environments who requires the simultaneous execution of actions. The MATB consists in four subtasks: tracking (*TRCK*), auditory monitoring (*COMM*), resource management (*RMAN*), and response to event onsets (*SYSM*).

The demand of the system monitoring task is monitoring the gauges and the warning lights by responding to the absence of the Green light, the presence of the Red light and monitors the four moving pointer dials for deviation from midpoint. The demand of manual control is simulated by the tracking task. The subject has to keep the cursor inside a squared target by moving the joystick. This task can be automated to simulate the reduced manual demands, for example if the subject became overloaded, the system would take the control. Subjects are also required to respond to a communication task. This task presents pre-recorded auditory messages at specific time intervals during the simulation. Not all of the messages are relevant to the operator. The goal of the COMM task is to determine which messages are relevant and to respond by selecting the appropriate radio and frequency on the communications task window. The demands of fuel management are simulated by the resource management task (RMAN). The goal is to maintain the fuel level of the main tanks at 2,500 (lbs) by turning on or off any of the eight pumps. Pump failures occur when they are red colored. Four performance indexes have been defined for the MATB; one index for each sub-task of the MATB. In particular, the index for the TRCK task has been defined by considering the complement of the ratio between the cursor's distance got by the subject and the maximum of this distance (fixed) from the center of the screen. The indexes of the COMM and SYSM tasks have been defined as a linear combination of accuracies in terms of correct answers (e.g., correct radio or frequency selected) and the complement of the ratio between the subject's reaction time and the maximum time for answering; then, the results have been multiplied for “100” in order to obtain a percentage. Finally, the index for the RMAN task has been defined as the mean value of the fuel's levels in the main tanks and then multiplied by “100.” In order to get a global *Performance Index*, the average of the previous indexes has been calculated. Additionally, two more indexes have been used in the analysis, the *Mean performance* (i.e., mean performance value between couple of consecutive sessions), and the *Performance Stability* (i.e., performance difference between couple of consecutive sessions).

### Experimental protocol

The subjects have been asked to practice and learn to execute correctly the MATB for three consecutive weeks (*WEEK_1, WEEK_2*, and *WEEK_3*). In total, they have taken part in 11 training sessions with a duration of 30 min each. Each training session consisted to execute the MATB under two different difficulty levels (EASY and HARD), who differed in terms of number and the rate of events and the required time to react to them. In the EASY condition, the subject had to attend only the tracking task, while in the HARD condition, all the sub-tasks were running with different timing and number of events. For example, the easy condition time-outs were 30 (s) for the radio communications, 10 (s) for the light scales, 15 (s) for the emergency lights, pump rates of 1,000 (lbs/min) and 500 (lbs/min) for the auxiliary and main tanks, respectively, and a total of 3 radio calls. Instead, the hard conditions was characterized by time-outs of 20 (s) for the COMM task, 5 (s) for the SYSM task, pump rates of 800 (lbs/min), and 600 (lbs/min) for the auxiliary and main tanks, respectively, and a total of 7 radio calls. The subjects had to execute each task condition twice during each training session. The only difference among the same difficulty level condition was the events' order. In fact, the types and the numbers of events have been kept the same for similar conditions. For example, if the first event of the hard1 condition was an emergency light, in the hard2 condition the first event was a pump failure. Also, the temporal order of the proposed conditions has been randomly selected to avoid expectation and habituation effects.

### Signals recording and processing

Electroencephalogram (EEG) has been recorded by a digital monitoring system (*ANT Waveguard system*) with a sampling frequency of 256 (Hz). All the 64 EEG electrodes have referred to both earlobes, grounded to the *AFz* channel and their impedances have been kept below 10 (kΩ). The EEG signal has been firstly band-pass filtered with a fifth-order Butterworth filter [low-pass filter cut-off frequency: 30 (Hz), high-pass filter cut-off frequency: 1 (Hz)], and then it has been segmented into epochs of 2 s (*Epoch length*), shifted of 0.125 s (*Shift*). *Independent Components Analysis* (Touretzky et al., 1996; ICA, Lee et al., 1999) has been performed to remove eyeblinks and eye saccades artifact, whilst for other sources of artifacts specific procedures of the EEGLAB toolbox have been used (Delorme and Makeig, 2004). In particular, three criteria have been applied to recognize artifacts. *Threshold criterion:* if the EEG signal amplitude exceed ±100 (μV), the corresponding epoch would be marked as *artifact*. *Trend criterion:* each EEG epoch has been interpolated in order to check the slope of the trend within the considered epoch. If such slope was higher than 3 (μV/s) the considered epoch would be marked as *artifact*. *Sample-to-sample difference criterion:* if the amplitude difference between consecutive EEG samples was higher than 25 (μV), it meant that an abrupt variation (no-physiological) happened and the EEG epoch would be marked as *artifact*. At the end, all the EEG epochs marked as *artifact* have been rejected from the EEG dataset with the aim to have an *artifact-free* EEG signal from which estimate the brain variations along the training period. All the previous mentioned values have been chosen following the guidelines reported Delorme and Makeig (2004). From the *artifact-free* EEG dataset, the *Power Spectral Density* (PSD) has been calculated for each EEG epoch using a *Hanning* window of the same length of the considered epoch (2 s length, that means 0.5 (Hz) of frequency resolution). The application of a *Hanning* window helped to smooth the contribution of the signal close to the extremities of the segment (epoch), improving the accuracy of the PSD estimation (Harris, 1978). Then, the EEG frequency bands have been defined accordingly with the *Individual Alpha Frequency* (IAF)-value estimated for each subject (Klimesch, 1999). Since the alpha peak is mainly prominent during rest conditions, the subjects have been asked to keep the eyes closed for a minute before starting with the experiment. Such condition has then been used to estimate the IAF-value specifically for each subject.

Finally, a spectral features matrix (*EEG channels x Frequency bins*) has been obtained in the frequency bands and EEG channels mainly correlated to learning processes, and not affected by *EEG bands transition effect* (Klimesch, 1999). In particular, the theta (IAF-6 ÷ IAF-2) and alpha bands (IAF-2 ÷ IAF+2) have been considered over the EEG frontal (AF7, AF3, AF8, AF4, F7, F5, F3, F1, Fz, F2, F4, F6, and F8) and parietal channels (P1, P3, P5, P7, Pz, P2, P4, P6, and P8). In the proposed study, the spectral features domain within which the asSWLDA had to select the most significant characteristics closely related to learning processes was of:

(1) EEG Channels × Frequency bins=25×17=425 (features)

### Machine—learning analysis

As stated previously, the idea presented in this work was to use a machine learning approach to track changes in the user's brain features along the training program. The expected result was to note a *plateau* of the classifier performance once the user's brain features (e.g., frontal and parietal theta and alpha EEG activations) become stable across consecutive training sessions, in other words, when the user might be “cognitively” trained. For doing this, the machine learning model should not suffer of performance decreasing over time. In other words, it should be generic enough to follow the evolution of the brain processes across a period of motor-cognitive training. In this regard, we used the *automatic-stop StepWise Linear Discriminant Analaysis* (asSWLDA, Aricò et al., 2016a,b), a modified version of the (SWLDA) algorithm, has been employed. With respect to the classical implementation of the SWLDA, the asSWLDA is able to select automatically the right number of features to consider into the classification model and at the same time, mitigating both *under*- and *over-fitting* problems. In particular, the asSWLDA starts by creating an initial model of the discriminant function, where the most statistically significant feature (within the spectral domain quote previously) is added to the model for predicting the target labels (*pval*_*ij*_ < α_*ENTER*_), where *pval*_*ij*_ represents the *p*-value of the *i-th* feature at the *j-th* iteration (in this case the first iteration). Then, at every new iteration, a new term is added to the model (if *pval*_*ij*_ < α_*ENTER*_). If there are not more features that satisfy this condition, a *backward* elimination analysis is performed to remove the least statistically significant feature (if *pval*_*ij*_ > α_*REMOVE*_). The standard implementation of the SWLDA algorithm uses α_*ENTER*_ = 0.05 and α_*REMOVE*_ = 0.1, and no constrains on the *Iteraction*_*MAX*_ (predefined number of iterations) parameter are imposed. In other words, the feature selection keeps going unless there are no more features satisfying the entry (α_*ENTER*_) and the removal (α_*REMOVE*_) conditions (Draper, 1998). However, the value of the *Iteration*_*MAX*_ parameter could affect the performance of the classifier (*underfitting* or *overfitting*), and this could be an important issue for the application of machine learning algorithm across different days (i.e., different training sessions). The optimum solution to these problems would be a criteria able to automatically stop the algorithm when the best number of features, *#Features*_*OPTIMUM*_, are added to the model such as: #*Features*_*UNDERFITTING*_ < #*Features*_*OPTIMUM*_ < #*Features*_*OVERFITTING*_. More the features added to the model are (number of iterations increases), more the significance (*p-value*) of the model (*pModel*) decreases (tending to zero) with a decreasing exponential shape (convergence of the model). Therefore, the asSWLDA define the model by finding the best trade-off between the number of features and the convergence of the model, that is automatically stop the algorithm in correspondence of the minimum distance of the *Conv(#iter)* function from the origin ([0,0]-point):

(2) $$\left||Conv(\#iter_{BEST})||\;=min||Conv(\#iter)|\right|$$

In other words, we sought the condition in which the following condition was satisfied (see Aricò et al., 2016a for further details):

(3) $$log_{10}\left(pModel\left(\#iter+1\right)\right)-log_{10}\left(pModel\left(\#iter\right)\right)=0$$

A two-class asSWLDA model has been used to select within the calibration EEG dataset the most relevant EEG spectral features to discriminate the task conditions (i.e., Easy vs. Hard). A 10-fold cross-validation have been performed by segmenting the entire dataset (e.g., merging of Easy 1—Easy 2, and Hard 1—Hard 2) in 10 parts. In this regard, such parts have been shuffled and then 9 parts have been used to calibrate the asSWLDA, while the remaining part to test it, exploring all the possible combinations (10 combinations in total). In this regard, the *Linear Discriminant Function* has been calculated for each testing dataset and the *Area Under Curve* (AUC)-values of the *Receiver Operating Characteristic* (ROC, Bamber, 1975) have been estimated to evaluate the performance of the classifier. We chose such kind of indicator, since its statistical property, in other words, the AUC of a classifier is equivalent to the probability that the classifier will rank a randomly chosen positive instance higher than a randomly chosen negative instance. This is equivalent to the Wilcoxon test of ranks (Fawcett, 2006). Two kind of analyses have been performed for each subject: *Intra*- and *Inter*-analysis. In the *Intra analysis* the calibrating and testing dataset have been taken within the same session (e.g., the first 90% of Easy and Hard data of Session X to calibrate the classifier, and the last 10% of Easy and Hard data of Session X to test it). On the contrary, for the *Inter analysis*, calibrating and testing dataset have been considered between consecutive sessions (e.g., the first 90% of Easy and Hard data of Session X for calibrating the classifier, and the last 10% of Easy and Hard data of Session X+1 to test it, and then the first 90% of Easy and Hard data of Session X+1 for calibrating the classifier, and the last 10% of Easy and Hard data of Session X to test it), and finally averaging the related AUC-values. To test our experimental hypothesis, we compared AUC-values related to *Intra analysis*, with respect to AUC-values related to *Inter analysis*. In fact, as stated previously, we expected that if the features of the subjects were changing over time, the Inter AUCs should be significantly lower than the Intra AUCs (i.e., within the same session the classifier should work at the best). On the contrary, if the Inter AUCs did not change significantly from Intra AUCs-values, it would mean that the features selected by the classifier were successful in both consecutive sessions. In other words, we should assume that the selected brain features remain stable across sessions. In this regard, we defined an index allowing to quantify the subject progresses from a cognitive point of view (*Cognitive Stability Index*). In particular, two paired two-tailed *t*-tests (α = 0.05) performed between each Intra related cross-validations AUC-values of consecutive sessions and the related Inter session have been performed, in order to quantify from a statistical point of view any difference between Intra and Inter related AUC-values. The *Cognitive Stability Index* (equation 1) has been defined as the average between such two *t*-values. We expected a decreasing trend of this index along sessions, since at the beginning of the training period the subject should not be able to accomplish correctly and automatically the proposed task (i.e., not trained), so the Intra and Inter related AUC-values should be significantly different (high *t*-values). Once the subjects became more confident (i.e., trained) with the task, the difference between Intra and Inter related values of consecutive sessions should tend to zero (i.e., not significant differences), that might represent stability in terms of brain activation patterns along the training sessions.

(4) $$\text{Cognitive Stability Index }\left(\text{n}\prime \right)=\frac{\left(\text{t}\left(\text{n}\right)+\text{t }\left(\text{n}+1\right)\right)}{2}$$

Where,

(5) t(n)=ttest(AUC(Intra(n)), AUC(Inter(n,n+1)))

(6) t(n+1)= ​ttest(AUC(Intra(n+1)), AUC(Inter(n,n+1)))

and *n* = {T1,T2,…,TX-1}; *X* = 12 sessions.

To investigate the trends and changes of the EEG signal throughout the training period (3 weeks), as signs of learning progress, and to obtain a robust statistic, the behavioral and physiological data have been analyzed in 6 representative training sessions (Kelly and Garavan, 2005). In particular, the 3 consecutive recording sessions of WEEK_1 (T1, T3, and T5, in T2 and T4 only behavioral (i.e., task performance) and neurophysiological data (i.e., AUC-values) have been recorded), the session of WEEK_2 (T7), and the last session of WEEK_3 (T12) have been considered. Since the subjects had never done the MATB in the past, instructions about how to execute the task have been provided on the first day of training (T1) before starting with the experiment. To be efficient, the instructional design was tailored to the aptitude of the subject in order to avoid that the effectiveness of the training was likely random (Kalyuga et al., 2003).

### Metric proposal for training level assessment

**Figure 2:**
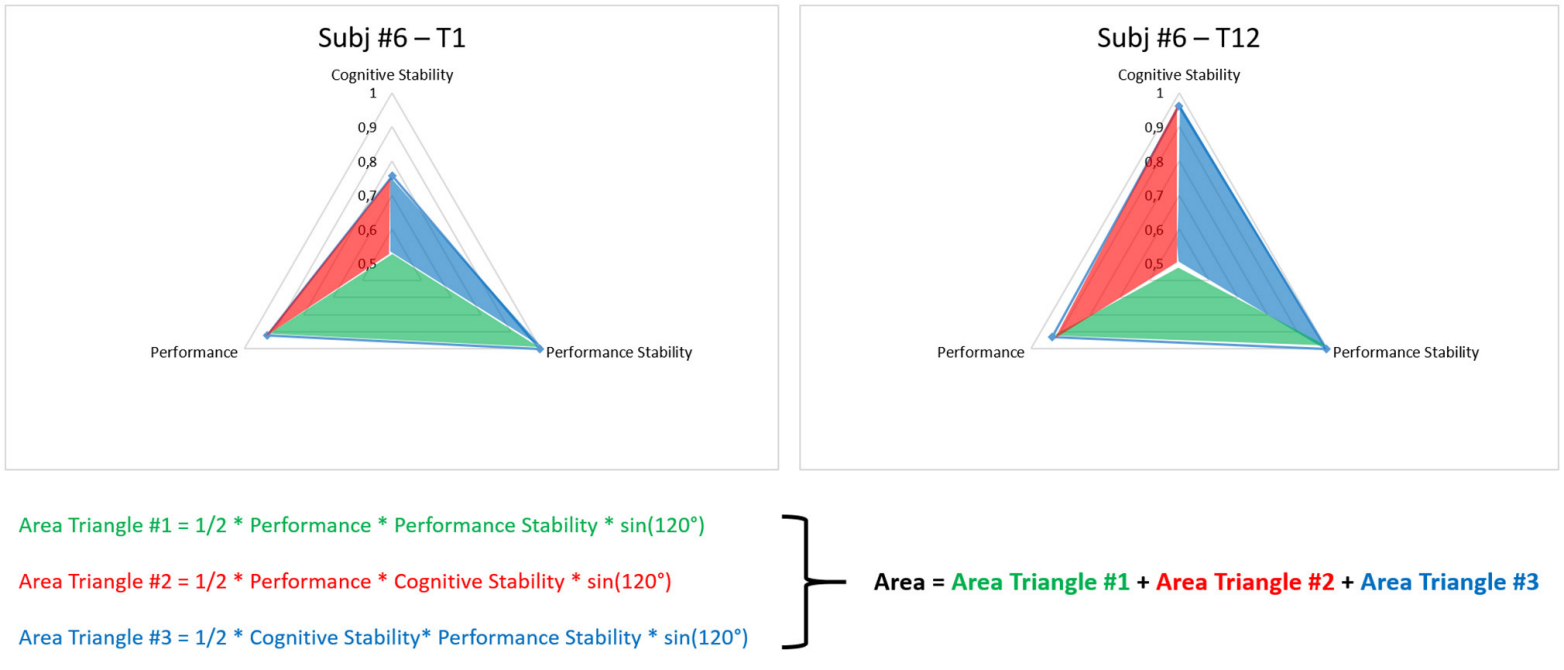
The images reports an example of triangle by which is possible to quantify the training level of the user. In particular, the area of the triangle, defined by considering the *Mean Performance, Performance Stability*, and *Cognitive Stability* as vertices, has been calculated as the sum of the areas of three sub-triangles (Area Triangle #1, Area Triangle #2, and Area Triangle #3). Such triangles were identified by the three indexes and the origin of the coordinate system.

In order to quantify the user's training level, a metric has been proposed and defined as combination of behavioral and neurophysiological data. In particular, three measures have been integrated to simultaneously take into account the level of task execution, and both the performance and cognitive stabilities over consecutive sessions. The three measures have been normalized with respect to their maximum values in order to have a domain of variation between 0 and 1. Then, they have been used to define a scalene triangle with the origin corresponding with its centroid (Figure 2). In particular, the vertexes of such a triangle (*A, B*, and *C*) have been defined by the values of the behavioral (*Mean Performance, Performance Stability*) and neurophysiological measures (*Cognitive Stability*) as distances with respect to the origin (centroid).

Therefore, such indexes identified three sub-triangles with the origin of the coordinate system, and the user's training level has been quantified by calculating the *Area* of the triangle as the sum of the areas of the sub-triangles:

(7) $$\begin{array}{l} Area=\;\frac{1}{2}ABsin\left(120{}^{\circ}\right)+\frac{1}{2}ACsin\left(120{}^{\circ}\right)\\ \;+\frac{1}{2}CBsin\left(120{}^{\circ}\right) \end{array}$$

Such an Area has then been normalized with respect to its maximum (Area MAX) corresponding to a triangle with all the sides equal to 1, as the following:

(8) $$Area\;MAX=\frac{3}{2}\sin\left(120{}^{\circ}\right)=1.299$$

As the AREA was closer to 1, as the user's training level was achieving the maximum level, as the three measures were reaching the maximum values (i.e., 1).

### Statistical analyses

#### Behavioral performance

A repeated measure ANOVA has been done on the performance index and *within* factor SESSION (6 levels: T1, T3, T5, T7, T9, and T12) with the aim to asses if significant increments happened, in terms of task execution, along the training period.

#### Cognitive stability index

A repeated measure ANOVA has been done on the Cognitive Stability Index and *within* factor CROSS-VALIDATION (6 levels: T1′, T3′, T5′, T7′, T9,′ and T12′) with the aim to asses if the subjects became trained, in terms of cognitive activations, along the training period.

#### Correlation between behavioral and neurophysiological measures

Pearson's correlation has been performed between the *Mean Performance* and *Cognitive Stability Index* with the aim to assess if the measures were coherent and could provide information related to the training.

#### Objective training assessment

A repeated measure ANOVA has been done on the Area data and *within* factor CROSS-VALIDATION (6 levels: T1′, T3′, T5′, T7′, T9,′ and T12′) with the aim to asses if the proposed metric could track and provide objective information about training level progresses along the different sessions.

## Results

### Behavioral performance

**Figure 3:**
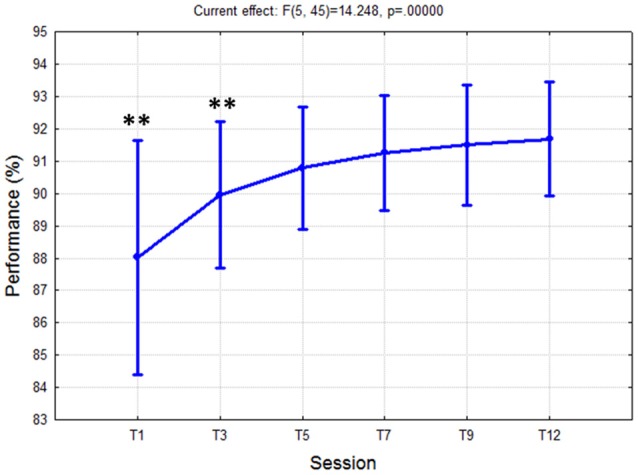
Task performance values over 3 weeks of training. The ANOVA showed a significant (*p* < 10^−5^) improvement of performance from T1 to T5, and then no differences were found between the rest of the sessions. Such results indicated that the subjects reached the task saturation in the session T5. “^**^” Means that the statistical significance level (*p*) is lower than 0.01. Vertical bars denote 0.95 confidence intervals (CI).

The ANOVA highlighted significant differences [*F*_(5, 45)_ = 14.25; *p* < 10^−5^] on task performance (Figure 3) across the different training sessions. In particular, the *post-hoc* test showed that from T5 the subjects improved significantly the level of their task performance (*p* < 10^−4^) with respect to T1 (first session), and then they kept such a high level of performance (about 91%) stable (i.e., no significant differences among consecutive sessions) until the end of the training period (T5÷T12). Therefore, in terms of task execution, the subjects could be retained trained since the session T5.

### Cognitive stability

**Figure 4:**
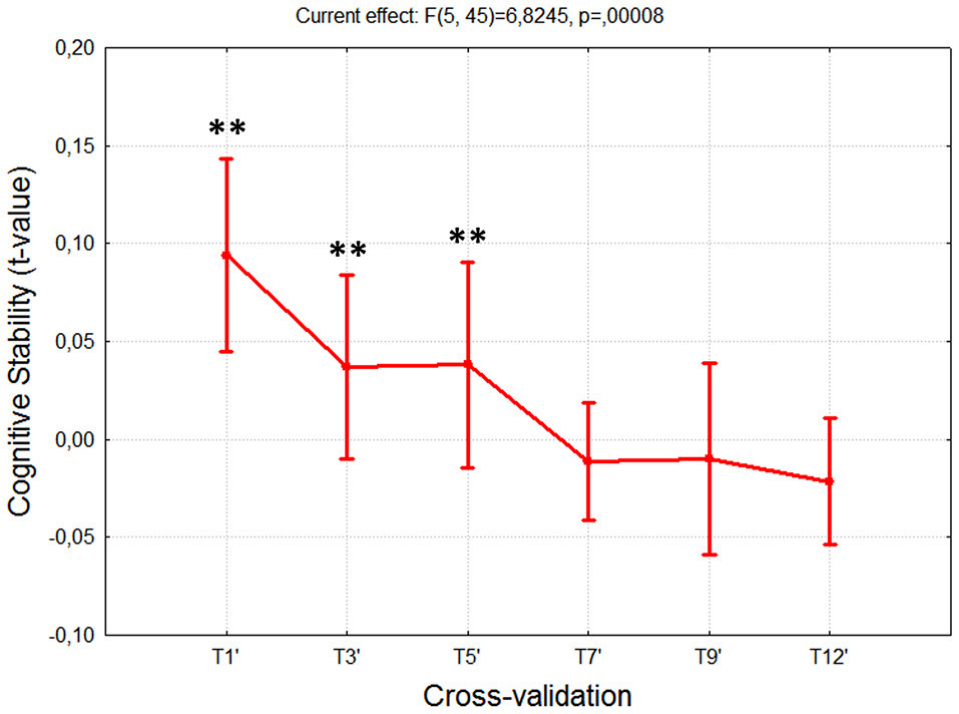
Cognitive Stability Index over 3 weeks of training. The ANOVA showed significant differences (*p* < 10^−4^) among the first sessions (T1÷T7), while no differences were found among the last ones (T7÷T12). Such results indicated that the subjects reached the cognitive stability in the session T7. “^**^” Means that the statistical significance level (*p*) is lower than 0.01. Vertical bars denote 0.95 confidence intervals (CI).

The results of the ANOVA on the *Cognitive Stability Index* reported significant difference along the training sessions [*F*_(5, 45)_ = 6.65; *p* = 0.0001]. As expected, the *post-hoc* test showed significant reductions, tending to zero, among the sessions T1÷T7, whereas no differences were found (all *p* > 0.57) from the session T7 to the last one (T12), since the differences, in terms of cognitive stability, were almost equal to zero (Figure 4).

**Figure 5:**
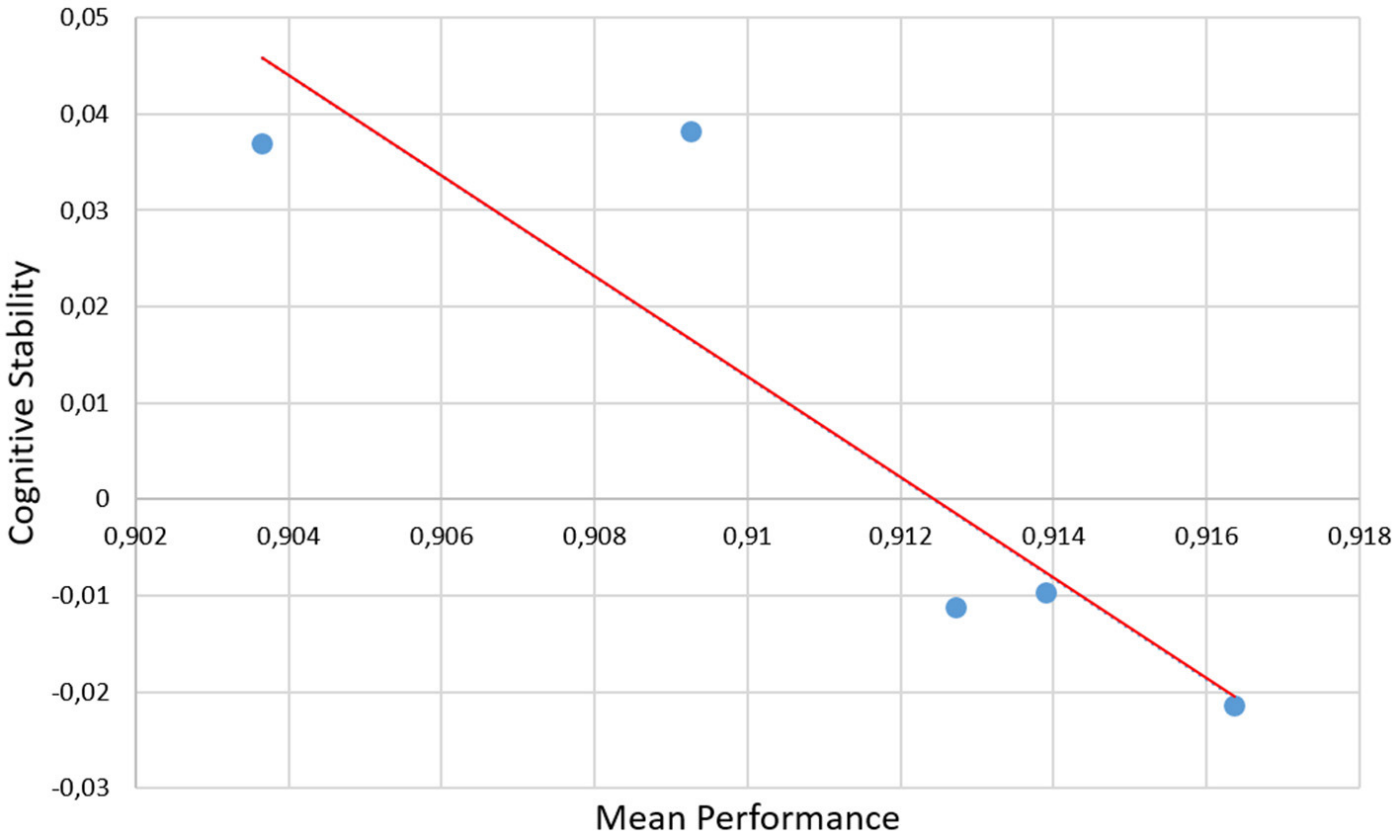
Scatter-plots of the correlation between the *Mean Performance* and the *Cognitive Stability*. The Pearson's analysis reported significant (*p* = 0.039) and high correlation (|*R*| > 0.89) between the measures, as demonstration that their integration could provide the Instructor objective data for the training assessment.

Furthermore, the correlations between the behavioral and neurophysiological measures have been investigated. In particular, Figure 5 shows the scatter-plot of the correlation between the *Mean Performance* and the *Cognitive Stability Indexes*. The Pearson's analysis reported significant (*p* = 0.039) and high correlations (|*R*| > 0.89) between the measures. In fact, more the task performance became stable, more the cognitive stability index tended to zero, that could mean no significant differences in terms of brain activation patterns when the participants becomes trained.

### Metric for training level assessment

**Figure 6:**
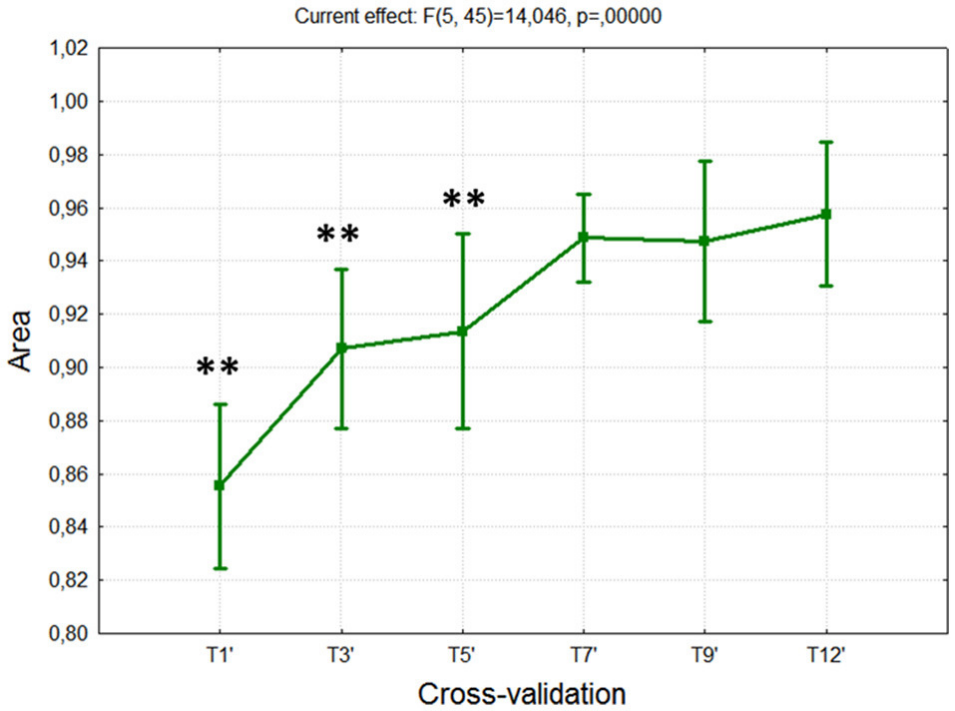
Training metric over 3 weeks of training. The proposed metric takes into account the level of task execution (performance), and both the behavioral and cognitive stabilities. The ANOVA showed significant differences (*p* < 10^−5^) among the first sessions (T1÷T7), while no differences were found among the last ones (T7÷T12). Such results indicated that the subjects reached a training stability in the session T7. “^**^” Means that the statistical significance level (*p*) is lower than 0.01. Vertical bars denote 0.95 confidence intervals (CI).

The ANOVA on the averaged Area reported (Figure 6) a significant increase across the training sessions [*F*_(5, 45)_ = 14.05; *p* < 10^−5^]. The *post-hoc* test showed that by considering both the behavioral and neurophysiological measures, the subjects exhibited significant differences (all *p* < 0.01) in the first part of the training period (T1÷T7), and then no more differences (all *p* > 0.51) over the last sessions (T7÷T12). In fact, across the training sessions the subjects improved their skills in terms of task execution, performance level and capability in retaining such a high level of performance (i.e., performance stability), and cognitive activations (i.e., cognitive stability).

**Figure 7:**
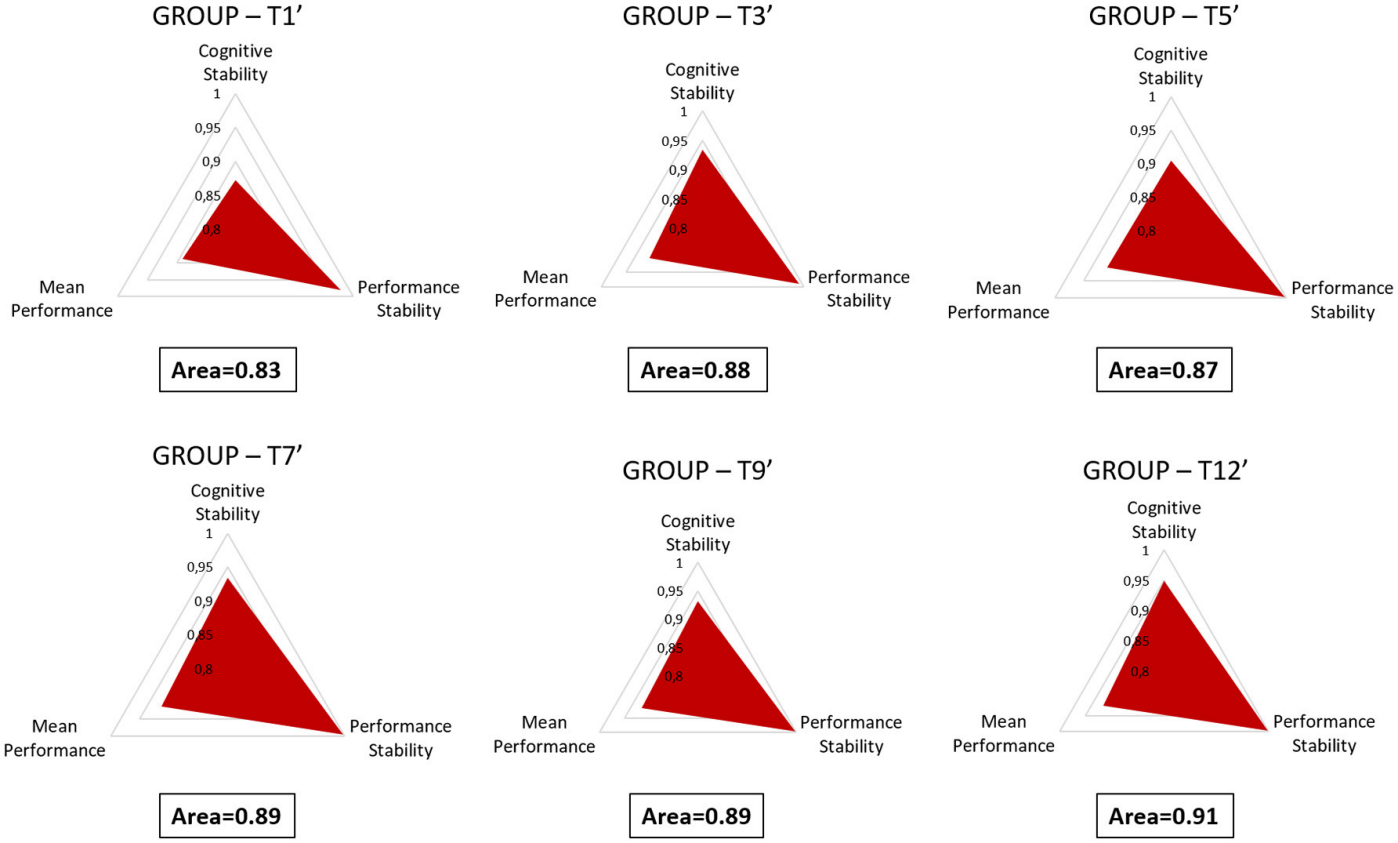
In order to quantify the users' training level along the training sessions, the behavioral and neurophysiological measures were integrated. Such measures were used to define the sides of a scalene triangle, and then the Area of such a triangle was calculated in each training session as measure of the training level. The Area was normalized with respect to its maximum with the aim to have as references of maximum training the value “1.”

In Figure 7, we have reported the Area values averaged across the subjects, along the considered training sessions in order to demonstrate how the experimental group kept improving their task and cognitive performance along the training period.

**Figure 8:**
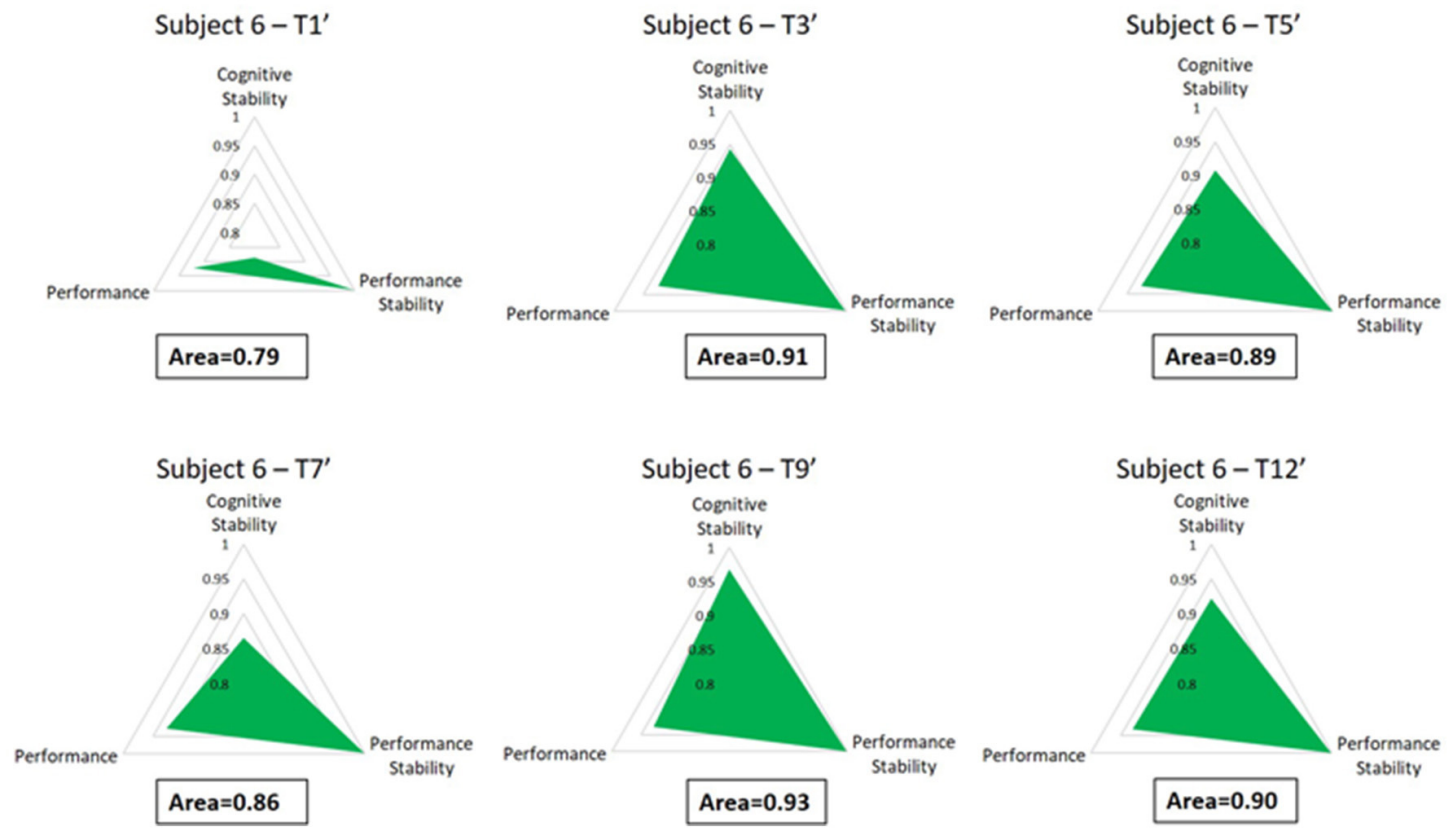
The figure reports the Areas of a representative subject along the considered training sessions. It is possible to appreciate how the Subject 6 improved its behavioral and cognitive skills from the beginning (T1) to the end of the training period (T12).

Figure 8 shows the advancement of the training metric (Area) for a representative subject (Subject 6). The gradual growth of the colored triangle is related to the training improvement along the considered sessions, as demonstration that by the proposed metric it should be possible to assess in each session the subject's training level, by considering “1” as maximum level.

## Discussion

The proposed work investigated the possibility to use a machine learning-based neurometric to provide additional and objective information regarding the progresses of a trainee throughout the training program on the base of the actual brain activations with respect to previous sessions. In fact, current machine learning methodologies generally aim to improve spectral and temporal features selection, reduce computational and time demand for the algorithm calibration (e.g., single trial calibration), or evaluate user's mental state and emotions. Furthermore, studies regarding learning evaluation usually highlighted the most evident changes on the considered neurophysiological parameter (e.g., event-related potentials, heart rate, specific EEG spectral power densities) between the first and last training session, without providing any objective measure to track learning progresses across the different training sessions, and to quantify the training level of the user in each session by comparing it with a maximum value.

Thus, the idea of our study was to use a machine learning algorithm in a different way and with the possibility of being applied over time, that is to compare different training sessions. In this regard, we selected the asSWLDA algorithm since it has been demonstrated to (i) being stable over time (no recalibration up to a month), and (ii) being robust to the problems of *under*- and *overfitting*, thus being able to select the features mainly linked to the considered cognitive phenomenon (Aricò et al., 2016a). The asSWLDA has been used to highlight changes in those brain features mostly linked to learning cognitive processes, and then its performance have been combined with the user's behavioral data to define a neurometric for the objective training assessment. In particular, the proposed neurometric takes into account the mean performance level achieved by the user (capability in executing correctly the task), the stability of the performance across different sessions (capability in maintaining high performance over time), and the stability of the brain activations across consecutive training sessions (capability in dealing with the task requiring the same amount of cognitive resources once it became automatic). By considering such aspects, we were able to provide a measure of the training level and to assess if the single user could be retained “trained” or not.

The analysis on the task performance (Figure 3) highlighted the existence of a learning effect. In fact, since the session T5 the subjects achieved a significantly higher (*p* < 0.001) level of performance than T1, and then they kept it stable throughout the rest of the sessions. In other words, no differences were found among the rest of the considered training sessions (T5÷12). Therefore, in terms of task execution the subjects seemed trained since the session T5. By the analysis of the EEG signal, the machine learning model was calibrated by selecting brain features within specific domains. In particular, the considered brain features were those mainly linked to the amount of information processing, decision making, and task difficulty (frontal theta EEG band), to the memory consolidation (parietal theta EEG band), to an improved access to the *Knowledge System* (KS), and more automatic actions (frontal alpha EEG band), and working memory load (parietal alpha EEG band). The analysis on the *Cognitive Stability Index* (neurophysiological metric) showed that (Figures 4) from the session T7, the brain activation patterns became stable until the end of the training, since significant differences were found among the first sessions (T1÷T7), while no differences were found among the last sessions (T7÷T12). We investigated the correlation between such measures with the aim to assess if they could provide coherent information about the users' training progress. The results (Figure 5) suggested that the Cognitive Stability Index and the Mean Performance showed significant (*p* = 0.039) and high correlation (|*R*| > 0.89). Therefore, they could be integrated to define a metric for an objective training assessment. In fact, we proposed a metric based on the both performance, and cognitive stability, and the level of task execution (*Mean Performance*). By such parameters, we could take into account the performance level of consecutive sessions (correct execution of the proposed task) and the behavioral and cognitive stability when dealing with it. The results showed that the proposed metric (*Triangle's Area*) was able to quantify the users' training level across the different sessions (Figure 6), by considering as “1” the maximum level of the metric, and they achieved a training stability since the session T7 (Figures 7, 8), since no statistical differences were found among the last sessions (T7÷T12).

At the moment the study presents some limitations. The first limitation is the size of the experimental group. This number is sufficient to highlight some significant statistical evidences, but of course, it needs to be enlarged to demonstrate the effectiveness of the proposed method. A second limitation is the proposed task. While the MATB is good for the analysis of the brain reactions while handling with multiple tasks, it could be reasonable to retain that training programs in realistic environments (e.g., pilots, controllers, or surgeons) could be different from those elicited by a laboratory task. Therefore, the results presented in this work have to be considered as a promising step for further and more ecological studies.

## Conclusions

In this paper, we proposed a methodology able to provide quantitative information about training progresses along the training sessions. In particular, we considered specific EEG rhythms coming from a deep literature review throughout a period of 3 weeks. In this regard, we used such brain features to calibrate a machine learning algorithm (i.e., asSWLDA), and to assess when the subjects reached a stability in terms of task execution (task performance) and brain activation patterns across the different sessions. The hypothesis was that, when the subject is untrained, the brain activations should differ any time he/she will perform the proposed task. On the contrary, if the subject is trained, the patter of brain activations should be almost stable across consecutive sessions. We highlighted such effects by means of a machine learning approach. In particular, we defined a neurophysiological parameter (*Cognitive Stability Index*) by integrating the performance of the classifier *within* the same session (Intra analysis) and *between* sessions (Inter analysis). Such information has been combined with the behavioral data to define a neurometric by which track and quantify the training level for each participant along the different sessions. The results highlighted the added-value of the proposed neurometric as complementary information to the standard performance evaluation, and stressed the importance of multi-modal training assessment for a more accurate training evaluation. In fact, different subjects could achieve the same task performance level, but requiring different amount of brain resources and showing different expertise (Borghini et al., 2017). Therefore, by the only task performance it would not be possible to obtain information in terms of cognitive activations and to assert possible differences between the subjects. Furthermore, an objective training assessment could provide useful data for the selection of the personnel or teams both in standard and extreme working contexts (Astolfi et al., 2012; Toppi et al., 2016).

## Author contributions

GB, PA, GD, and NS: EEG recordings, training neurophysiological characterization, data analysis, and paper writing. GB, PA, GD, AC, NT, and FB: experimental protocol design, methodology development. GB, PA, GD, AB, and NT: validation of the experimental procotol, recruiting of volunteers, providing experimental facilities. AC, MH, AB, NT, and FB: methodology, results and paper checking.

### Conflict of interest statement

The authors declare that the research was conducted in the absence of any commercial or financial relationships that could be construed as a potential conflict of interest.
